# Commentary: Rehebbilitating Memory

**DOI:** 10.3389/fnbeh.2016.00078

**Published:** 2016-04-22

**Authors:** Pascale Gisquet-Verrier, David C. Riccio

**Affiliations:** ^1^Neuro-PSI, Université Paris-Sud, Centre National de la Recherche Scientifique UMR 9197, Université Paris-SaclayOrsay, France; ^2^Department of Psychological Sciences, Kent State UniversityKent, OH, USA

**Keywords:** consolidation, reconsolidation, reactivation, retrieval, morphological changes, protein synthesis inhibitors, state dependency, malleability

In 2015, two papers using very different procedures presented strong evidence indicating that amnesia induced by protein synthesis inhibitor did not prevent memory formation, challenging the classical consolidation hypothesis (Gisquet-Verrier et al., 2015; Ryan et al., 2015). Both of them proposed that amnesia resulted from retrieval difficulties. In their recent review, Ryan and Tonegawa (2016) went further and proposed that retrieval difficulties were due to the absence of training-induced morphological changes induced by protein synthesis inhibition. However, as explained in the present comment, there is evidence arguing against this position.

According to the traditional consolidation hypothesis which has dominated the literature concerning memory formation for more than 50 years, newly acquired information is progressively encoded into a stable long-term memory. Most of the support for this hypothesis relies on retrograde amnesia produced by various treatments delivered just after a training episode. Amnesic agents initially were severe treatments (e.g., electroconvulsive shock, cortical spreading depression, hypothermia…) that disrupted the normal functioning of the brain during this stabilization process. Later, protein synthesis inhibitors (PSI) became the most commonly used amnesic agents as they could be targeted directly into brain areas assumed critical for specific aspects of the memory, such as the amygdala for fear conditioning (Nader et al., 2000). The current version of the consolidation hypothesis now assumes that consolidation is achieved through processes requiring new protein synthesis.

Historically, the consolidation hypothesis has been challenged by a series of studies showing that memory can be recovered following the exposure to a variety of “reminder” treatments (Lewis, 1979; Miller and Matzel, 2006). These studies were not consistent with a consolidation hypothesis postulating that interrupting the stabilization processes should lead to a permanent loss of the memory, and rather suggested that amnesia resulted from retrieval difficulties (Lewis, 1979). However, the finding of long-term potentiation, which fit well with the consolidation hypothesis, became the dominant explanation of memory consolidation at a cellular level (Abel and Lattal, 2001) and the behavioral criticisms were largely ignored.

In a recent series of experiments Tonegawa and his colleagues (Ryan et al., 2015), using sophisticated techniques involving learning-dependent cell labeling, were able to show that a memory survives amnesia. They demonstrated that while a protein synthesis inhibitor delivered immediately after the conditioning episode induced amnesia; optogenetic activation of the engram cells unexpectedly resulted in memory retrieval. In other words, PSI rendered rats amnesic but the memory was “rescued” by a direct activation of the memory engram cells. This result demonstrated that protein synthesis was not required for the formation of new memory, challenging the consolidation hypothesis. In the same study, the authors demonstrated that inhibition of protein synthesis delivered within the brain also prevented enhanced dendritic spine density and synaptic strength normally observed in the engram cells engaged in the fear memory. Based on this finding, Tonegawa and colleagues proposed that the primary role of training-induced morphological changes is to participate in memory retrieval by allowing natural recall cues access to the engram cells. In their recent review paper, Ryan and Tonegawa (2016) returned to these results by placing a greater emphasis on the role protein-induced morphological changes may have in retrieval processes.

However, their position seems inconsistent with two sets of results.

It is well known that amnesia can be induced by treatments delivered both after conditioning and after memory reactivation (Lewis, 1979; Nader et al., 2000) and Tonegawa's team replicated this finding (Ryan et al., 2015). In the case of post-reactivation amnesia, the morphological changes thought to be induced by initial training normally took place after training, as rats demonstrated substantial levels of freezing during memory reactivation, indicating that retrieval processes were in place at that time. However, rats treated with PSI after reactivation were unable to express fear to the training context on the next day. Accordingly, either the morphological changes promoted by initial training were abolished by the post-reactivation PSI injection (which seems unlikely), or their difficulties in expressing fear arose from a source other than a lack of morphological changes.

Another set of studies indicates that retrograde amnesia can be alleviated by pretest exposure to various reminder cues including the amnesic treatment itself (Lewis, 1979; Riccio et al., 2006). It has been shown that, re-exposure to the PSI agent can reverse amnesia resulting from either post-training or post-reactivation PSI administration, independently of whether the treatment was administered systemically or intracerebrally (Bradley and Galal, 1988; Briggs and Olson, 2013; Gisquet-Verrier et al., 2015). These results complement the findings from the Tonegawa lab in showing that the formation of new memory does not require de novo protein synthesis. and that retrograde amnesia results from a deficit of memory retrievability rather than disruption of consolidation. The issue raised here only concerns the origins of memory retrieval difficulties. For Tonegawa, these difficulties are due to the PSI preventing training induced morphological changes. However, evidence that amnesia can be reversed by reinstating the internal state induced by PSI shortly before the retention test reveals that retrieval can be achieved even when the morphological changes due to conditioning were prevented by PSI.

We proposed an alternative retrieval interpretation to that of Ryan and Tonegawa based on a combination of memory malleability and state-dependency (Gisquet-Verrier et al., 2015). Due to the malleability of active memory (i.e., after training and reactivation), the internal state induced by PSI is integrated into the initial memory. Subsequent retrieval difficulties would thus be due to the absence of this salient cue at the time of testing, an effect which can be overcome by reinstating the internal state before the retention test. Based on the findings described above, *de novo* protein synthesis is neither required for memory formation nor retrieval and this leaves open the question concerning the role of the protein dependent morphological changes taking place shortly after training.

More generally, it seems clear, as Ryan and Tonegawa (2016) have pointed out, that retrieval oriented interpretations of amnesia offer new views on memory, suggesting that it is now timely to revisit the consolidation hypothesis.

## Author contributions

All authors listed, have made substantial, direct and intellectual contribution to the work, and approved it for publication.

## Funding

PG wishes to thank the Térèse and René Planiol foundation which provided a financial support.

### Conflict of interest statement

The authors declare that the research was conducted in the absence of any commercial or financial relationships that could be construed as a potential conflict of interest.
